# MuTATE—an R package for comprehensive multi-objective molecular modeling

**DOI:** 10.1093/bioinformatics/btad507

**Published:** 2023-09-09

**Authors:** Sarah G Ayton, Víctor Treviño

**Affiliations:** Tecnologico de Monterrey, Escuela de Medicina y Ciencias de la Salud, Monterrey, Mexico; Tecnologico de Monterrey, Escuela de Ingeniería y Ciencias, Monterrey, Mexico; Tecnologico de Monterrey, Escuela de Medicina y Ciencias de la Salud, Monterrey, Mexico; Tecnologico de Monterrey, The Institute for Obesity Research, Monterrey, Mexico

## Abstract

**Motivation:**

Comprehensive multi-omics studies have driven advances in disease modeling for effective precision medicine but pose a challenge for existing machine-learning approaches, which have limited interpretability across clinical endpoints. Automated, comprehensive disease modeling requires a machine-learning approach that can simultaneously identify disease subgroups and their defining molecular biomarkers by explaining multiple clinical endpoints. Current tools are restricted to individual endpoints or limited variable types, necessitate advanced computation skills, and require resource-intensive manual expert interpretation.

**Results:**

We developed Multi-Target Automated Tree Engine (MuTATE) for automated and comprehensive molecular modeling, which enables user-friendly multi-objective decision tree construction and visualization of relationships between molecular biomarkers and patient subgroups characterized by multiple clinical endpoints. MuTATE incorporates multiple targets throughout model construction and allows for target weights, enabling construction of interpretable decision trees that provide insights into disease heterogeneity and molecular signatures. MuTATE eliminates the need for manual synthesis of multiple non-explainable models, making it highly efficient and accessible for bioinformaticians and clinicians. The flexibility and versatility of MuTATE make it applicable to a wide range of complex diseases, including cancer, where it can improve therapeutic decisions by providing comprehensive molecular insights for precision medicine. MuTATE has the potential to transform biomarker discovery and subtype identification, leading to more effective and personalized treatment strategies in precision medicine, and advancing our understanding of disease mechanisms at the molecular level.

**Availability and implementation:**

MuTATE is freely available at GitHub (https://github.com/SarahAyton/MuTATE) under the GPLv3 license.

## 1 Introduction

Cancer subtyping and molecular modeling is increasingly important in precision medicine, as it enables identification and administration of targeted therapies based on a patient’s cancer subtype (Ayton *et al.* 2022, GBD 2019 Adolescent Young Adult Cancer Collaborators 2022). However, translating complex disease subtypes to the clinical setting remains a challenge (Adlung *et al.* 2021, Engelhardt and Michor 2021, Yung *et al.* 2021), particularly when considering the multiple clinical endpoints that define patient health (Banegas-Luna *et al.* 2021, Yoon *et al.* 2022). Traditional machine-learning (ML) approaches have identified disease subtypes, but they are challenging to interpret and have limited capacity to address multi-objective problems. Manual review has synthesized ML results into clinically interpretable models but is subjective and time consuming (Wang *et al.* 2020, Couckuyt *et al.* 2022).

Decision trees (Breiman *et al.* 1984) offer a clinically interpretable ML model, but have not been adequately adapted to address the multi-objective problem in complex molecular data. M5P (Quinlan 1993, Zhang *et al.* 2019), multi-target boosting (Schapire *et al.* 1999, Alfaro *et al.* 2013), multi-target random forest (Meinshausen 2005, Meinshausen *et al.* 2018), and multi-target decision tree (Zhou 2012) are decision tree methods designed to handle multiple continuous outcomes. Some convert categorical variables to continuous (Quinlan 1993, Zhou 2012, Zhang *et al.* 2019), while others handle multiple outcome variable types but not simultaneously in one model (Schapire *et al.* 1999, Meinshausen 2005, Meinshausen *et al.* 2018, Alfaro *et al.* 2013). These methods have limited capacity to handle outcome variable types, do not have options for target variable weights, and subsequently struggle with overfitting, limited interpretability, and scalability. Ensemble approaches achieve improved accuracy but do so at the expense of model explainability and are unable to derive comprehensive molecular signatures for clinical use. These approaches lose outcome explainability and do not generalize to clinical problems (Ho 1995, Breiman 1996, Friedman 2001, Wang and Sebag 2012, Jeong *et al.* 2020, Xu *et al.* 2020).

To address these challenges, we propose Multi-Target Automated Tree Engine (MuTATE), a novel multi-objective decision tree algorithm R package, which can produce clinically interpretable prognostic models of disease. This ML method adapts the classic decision tree framework to the multi-objective problem, allowing for the identification of comprehensive clinical subgroups that may benefit from different treatments.

## 2 Software implementation and features of MuTATE

MuTATE is an R-based software for subtype and molecular signature discovery that enables researchers to (i) quantitatively evaluate existing manually derived and synthesized architectures, (ii) perform hyperparameter tuning, (iii) train and test multi-outcome models, and (iv) enables users to develop a clinically interpretable and meaningful molecular model of complex disease that considers multiple clinical outcomes using their own datasets. It implements several partitioning methods in both stepwise and lookahead approaches that have been tested for inferring biomarker importance in describing complex disease heterogeneity across multiple clinical outcomes. Hyperparameter tuning uses grid search to assess model performance under user-specified parameter combinations, which enables quantitative parameter selection following tuning on training data. Pre-trained models can be used directly on molecular data for simultaneous biomarker and subtype discovery. Multi-target tree models can also be re-trained and tested on new real or synthetic data (e.g. assessing the translation of molecular signatures from one cancer to another, assessing molecular signatures in the same cancer across distinct populations, etc.) and can model a range of complex disease outcomes and outcome types (e.g. simultaneously classifying survival outcomes, disease progression, treatment response, etc.). Any user can easily develop novel comprehensive decision tree architectures, benefiting from parameter tuning, and training and testing procedures. The models generated are not only comprehensive and address multiple clinical outcomes but are also easy to interpret for use in a clinical setting.

**Figure 1.**
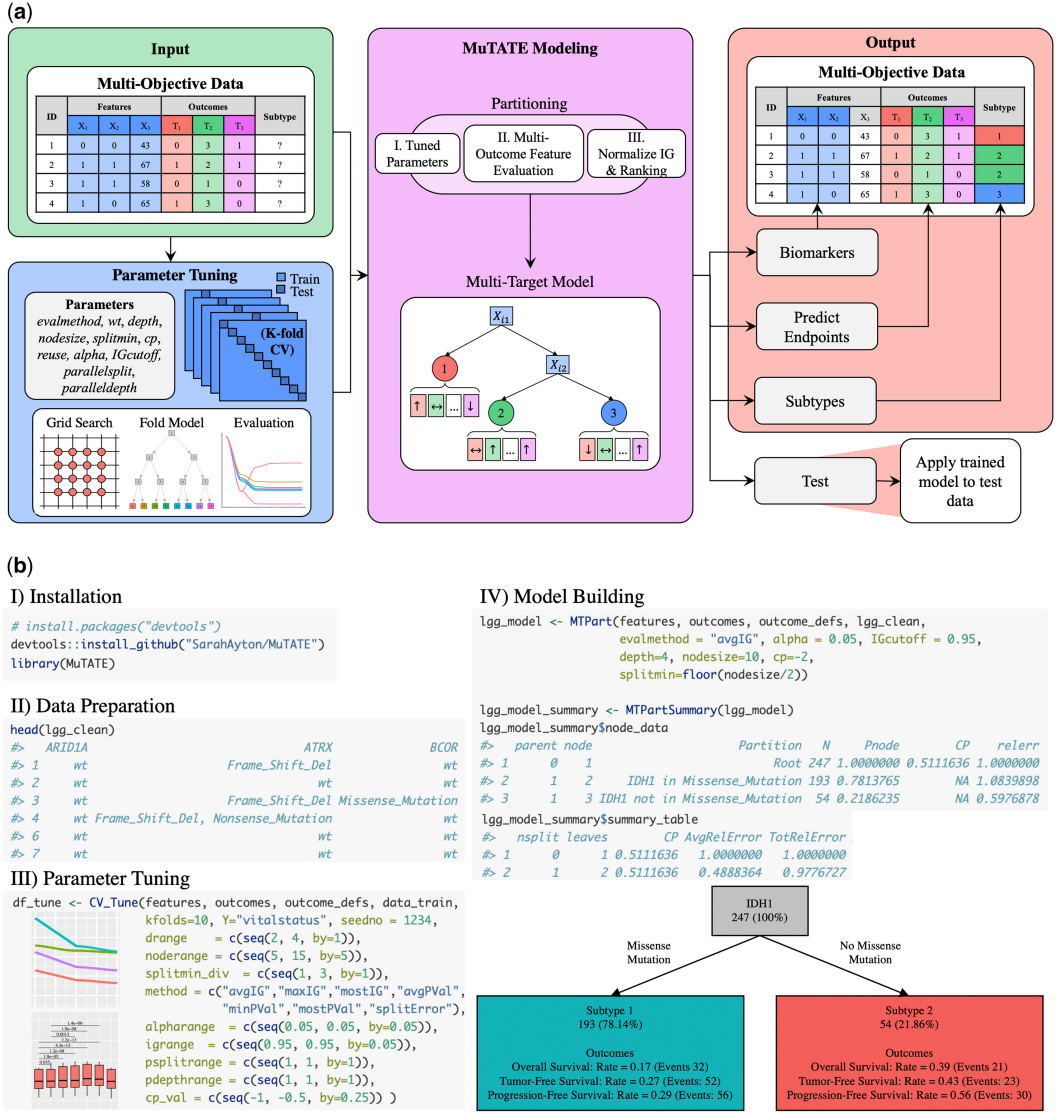
The MuTATE package and overview of the analytic process. Multi-objective data are input with several features and outcomes; dimensionality reduction should be performed prior to modeling. Using the CV_Tune function, the user can perform parameter tuning to assess potential model parameters and select the best performing set to perform MuTATE modeling, training the model with MTPart and using PlotTree for visualization. The trained model identifies subtypes and biomarkers and predicts endpoints for evaluation against observed values. The trained model may be applied to test data using MTTest. By providing explainable and quantitative molecular modeling across the panorama of clinical outcomes of interest, MuTATE enables enhanced, comprehensive screening, which can parse out and predict disease heterogeneity to identify patients who will benefit from different treatment plans, thereby improving health. This is contrasted with the existing standard of care, which does not comprehensively personalize treatment to maximize health, resulting in unpredictable clinical outcomes and overall health

Figure 1 provides an overview of model development using MuTATE. It is accessible without advanced ML knowledge due to its comprehensive tutorial and function documentation, which provide the user with a guide and a variety of options at each step of model development. As each research question and dataset is unique, our software package allows users to define their outcome variables (i.e. categorical, continuous, event rate, and time to event variable types) of interest and tune parameters with robust cross-validation and grid search by simply modifying function parameters, bypassing the need for extensive coding. We also include a function to explore model performance and enable quick and user-friendly visualization of constructed models. MuTATE provides explainable, quantitative, and user-friendly molecular modeling across the multiple clinical outcomes of interest in any dataset. This enables researchers to perform enhanced, comprehensive screening to parse out and predict disease heterogeneity and identify patients who will benefit from different treatment plans. By contrast, the existing standard of care is based on limited existing manually derived models built for a narrow range of the patient population and does not comprehensively personalize treatment to maximize health. Consequently, clinical outcomes and overall health can be unpredictable, especially in minority and other under-served and vulnerable populations. MuTATE has the potential to drive scientific discovery and improve the utility of decision trees in clinical and population health.

MuTATE automates feature evaluation, ranking, and partitioning by binarizing features, evaluating them against all targets, and forming rules across targets to create partitions or leaves. Consideration of multiple targets and target weights during model construction reduces greed and enhances subtypes and biomarkers discovery. Information gain (IG), the difference between parent and child node loss [continuous: deviance, categorical: Gini index, count: likelihood ratio (LR) test, and survival: exponential scaling with LR test] evaluates partitions during tree construction. The proportion IG is standardized for each target, enabling target comparisons. MuTATE provides several partitioning options (specified by evalmethod), including average multi-target IG (avgIG), highest IG in any target (maxIG), meaningful IG (≥IGcutoff) in the most targets (mostIG), lowest average *P*-value of statistically significant (≤α) IG (avgPVal), lowest *P*-value weighted by number of targets with significant IG (minPVal), significant IG in the most targets (mostPVal), or a subtree lookahead examining multi-target error (splitError). The package offers additional parameter flexibility, including options for the user to specify target weights wt, model depth *d*, minimum node size for partitioning nodesize, minimum leaf size splitmin, the complexity parameter cp (which applies a penalty), if features can be reused in subsequent partitions reuse, the alpha level alpha, the IG cutoff value IGcutoff, and number of parallel partitions parallelsplit and depth of subtrees paralleldepth to consider in splitError partitioning. MuTATE mitigates overfitting limitations by utilizing standardized proportion IG for partition evaluation across multiple targets. It offers parameter flexibility for model customization, including model depth, node and leaf sizes, and enables model training on one dataset and testing on another, while employing *k*-fold cross-validation for performance assessment.

MuTATE is an R (≥4.2) package available for download and installation from GitHub, which can be done directly in R using the install_github function from the devtools package (Wickham *et al.* 2022). MuTATE is licensed under the GPLv3 License. Release 1.0 is available from GitHub at https://github.com/SarahAyton/MuTATE and in R using install_github (“Sarah Ayton/MuTATE”). We showcase the MuTATE process in a tutorial, which enhances usability without prior coding knowledge. The tutorial guides the user through data pre-processing and parameter tuning, training a multi-target tree model, performing model evaluation, testing, and visualization. The tutorial is available at https://github.com/SarahAyton/MuTATE/blob/main/MuTATE_Tutorial.pdf.

## 3 Results, discussion, and conclusion

MuTATE is a R package for novel multi-objective decision tree generation that provides a comprehensive solution for automated and interpretable molecular modeling in precision medicine. It enables the identification of comprehensive clinical subgroups and their defining molecular biomarkers by explaining multiple clinical endpoints. Traditional ML approaches have limited interpretability across clinical endpoints, necessitate advanced computation skills, and require resource-intensive manual expert interpretation. MuTATE eliminates the need for manual synthesis of multiple non-explainable models, making it highly efficient and accessible for bioinformatics and clinicians.

MuTATE has the potential to transform biomarker discovery and subtype identification, leading to more effective and personalized treatment strategies in precision medicine. Its flexibility and versatility make it applicable to a wide range of complex diseases, including cancer. With the availability of the MuTATE R package, researchers and clinicians have access to a tool that can provide comprehensive molecular insights for precision medicine, improve therapeutic decisions, and advance our understanding of disease mechanisms at the molecular level. MuTATE offers a solution to the challenges of traditional ML approaches and manual review in comprehensive multi-objective molecular modeling. It provides an efficient and accessible tool for bioinformaticians and clinicians and has the potential to transform the field of precision medicine by enabling comprehensive molecular signature and subtype identification for effective and personalized treatment strategies.

## Data Availability

The data underlying this article are available in its associated online repository.
